# Exploring sources of insecurity for Ethiopian Oromo and Somali women who have given birth in Kakuma Refugee Camp: A Qualitative Study

**DOI:** 10.1371/journal.pmed.1003066

**Published:** 2020-03-24

**Authors:** Amber Trujillo Lalla, Katherine Farrell Ginsbach, Naomi Penney, Arsity Shamsudin, Rahul Oka

**Affiliations:** 1 University of New Mexico School of Medicine, Albuquerque, New Mexico, United States of America; 2 Eck Institute of Global Health, University of Notre Dame, Notre Dame, Indiana, United States of America; 3 Indiana University–Purdue University, Indianapolis, Indiana, United States of America; 4 Community Member, Kakuma Refugee Camp, Turkana, Kenya; 5 Anthropology Department, University of Notre Dame, Notre Dame, Indiana, United States of America; Columbia University Mailman School of Public Health, UNITED STATES

## Abstract

**Background:**

According to the United Nations High Commissioner for Refugees, 44,000 people are forced to flee their homes every day due to conflict or persecution. Although refugee camps are designed to provide a safe temporary location for displaced persons, increasing evidence demonstrates that the camps themselves have become stressful and dangerous long-term places—especially for women. However, there is limited literature focused on refugee women’s perspectives on their insecurity. This qualitative study sought to better understand the ways in which women experienced insecurity at a refugee camp in Kenya.

**Methods and findings:**

Between May 2017 and June 2017, ethnographic semi-structured interviews accompanied by observation were conducted with a snowball sampling of 20 Somali (*n =* 10) and Ethiopian Oromo (*n =* 10) women, 18 years and older, who had had at least 1 pregnancy while living in Kakuma Refugee Camp. The interviews were orally translated, transcribed, entered into Dedoose software for coding, and analyzed utilizing an ethnographic approach. Four sources of insecurity became evident: tension between refugees and the host community, intra- or intercultural conflicts, direct abuse and/or neglect by camp staff and security personnel, and unsafe situations in accessing healthcare–both in traveling to healthcare facilities and in the facilities themselves. Potential limitations include nonrandom sampling, the focus on a specific population, the inability to record interviews, and possible subtle errors in translation.

**Conclusions:**

In this study, we observed that women felt insecure in almost every area of the camp, with there being no place in the camp where the women felt safe. As it is well documented that insecure and stressful settings may have deleterious effects on health, understanding the sources of insecurity for women in refugee camps can help to guide services for healthcare in displaced settings. By creating a safer environment for these women in private, in public, and in the process of accessing care in refugee camps, we can improve health for them and their babies.

## Abstract 2

In a qualitative study, Amber Lalla and colleagues explore sources of insecurity for Ethiopian Oromo and Somali women who have given birth in Kakuma Refugee Camp.

## Author summary

**Why was this study done?:**

- Every day 44,000 people are forced to flee their homes due to conflict or persecution.
- Refugee camps are designed to provide a safe temporary location for displaced persons.
- Increasingly, evidence demonstrates that the camps themselves have been stressful and dangerous long-term places—especially for women.
- In this study, we sought to understand the ways in which women experienced insecurity while at a refugee camp in Kenya (Kakuma Refugee Camp).

**What did the researchers do and find?:**

- We interviewed 20 Somali and Ethiopian Oromo women who were 18 years and older who had had at least 1 pregnancy while living in Kakuma Refugee Camp.
- The interviews were translated, transcribed, and entered into Dedoose software, where they were coded.
- Findings included that women felt sources of insecurity stemmed from tension between refugees and the host communities, conflicts within the refugee community, and direct abuse and neglect by camp staff and security personnel.
- Women felt endangered traveling to health facilities, and this fear did not subside once within the health facility as they felt they were put in additional danger from inadequate and biased treatments.

**What do these findings mean?:**

- Many problems of insecurity and vulnerability stem from the lack of power refugees have in their own lives.
- Understanding the sources of insecurity that are faced by women in refugee camps can help to guide services for healthcare in displaced settings.
- By creating a safer environment for women in private, in public, and in accessing healthcare in refugee camps, we can improve health for them and their babies.
- Potential limitations of this study include the inability to obtain a random sample, the study’s focus on a specific population, the inability to record interviews, and the possible subtle errors in exact translation.

## Introduction

Political and economic insecurity is rising all over the world. According to the United Nations High Commissioner for Refugees (UNHCR), at the height of the current refugee crisis in 2018, there were approximately 70.8 million displaced persons, with an average of 25 people newly displaced every minute [1]. The increased number of displaced persons has amplified the crowding in refugee settings, contributing to a new source of economic and political insecurity for refugees. With an anticipated increase in births occurring in refugee and displaced persons environments, it is important to fully understand how these environments affect women. Refugee camps, generally located within resource-poor environments populated by local host communities that are themselves marginalized and impoverished, are a paradigm of such environments, making them a unique location for understanding the relationships between general insecurity and well-being [2].

In this paper, we describe the “political economy of insecurity,” defined as the political and economic environments that create an insecure environment for refugees. The political environment of a refugee camp is unique in that there are many overarching entities governing the site (UNHCR, the host country, nongovernmental organizations, etc.), leaving the refugees with little to no political power in their environment. The security and safety of refugees within refugee camps is a shared mandate of the government of the host country and UNHCR and is a required condition of the “humanitarian space” [3]. However, despite the presence of police and security teams, insecurity in refugee camps is still a major concern leading to both structural and physical violence [4]. The political environment also plays a role in the economic environment, as the governing organizations also can dictate the types of employment refugees can and cannot obtain while living in the camps. Many camps are not “integrated,” meaning the refugees cannot seek employment in the host community. The emergent political economy of insecurity in turn has differential impacts on refugee individuals and groups that differ in terms of factors such as assets/income, location within the camp, marital status, and—crucially as various studies have noted—sex [5–7].

Refugee women face unique challenges due to the hostile environment caused by increased insecurity. Refugee women are natural targets for various types of sexual and gender-based violence (SGBV) such as rape, trafficking, trading sex for food, forced early marriage, and female circumcision [5]. Previous literature has further indicated how increased exposure to constant violence has led to a constant fear in everyday life for encamped refugee women [6,7]. Therefore, the political economy of insecurity creates a high-stress environment and further acts as an overarching social stressor especially for refugee women.

Living with constant fear of crime or having experienced SGBV has been linked to self-reported poor health outcomes [8,9]. Other studies have proposed that psychological stress is an important mediator between insecure environments or exposure to violence and health outcomes [10]. However, there is minimal research focusing on insecurity for women in encamped refugee settings, specifically on understanding the sources of insecurity.

**Fig 1:**
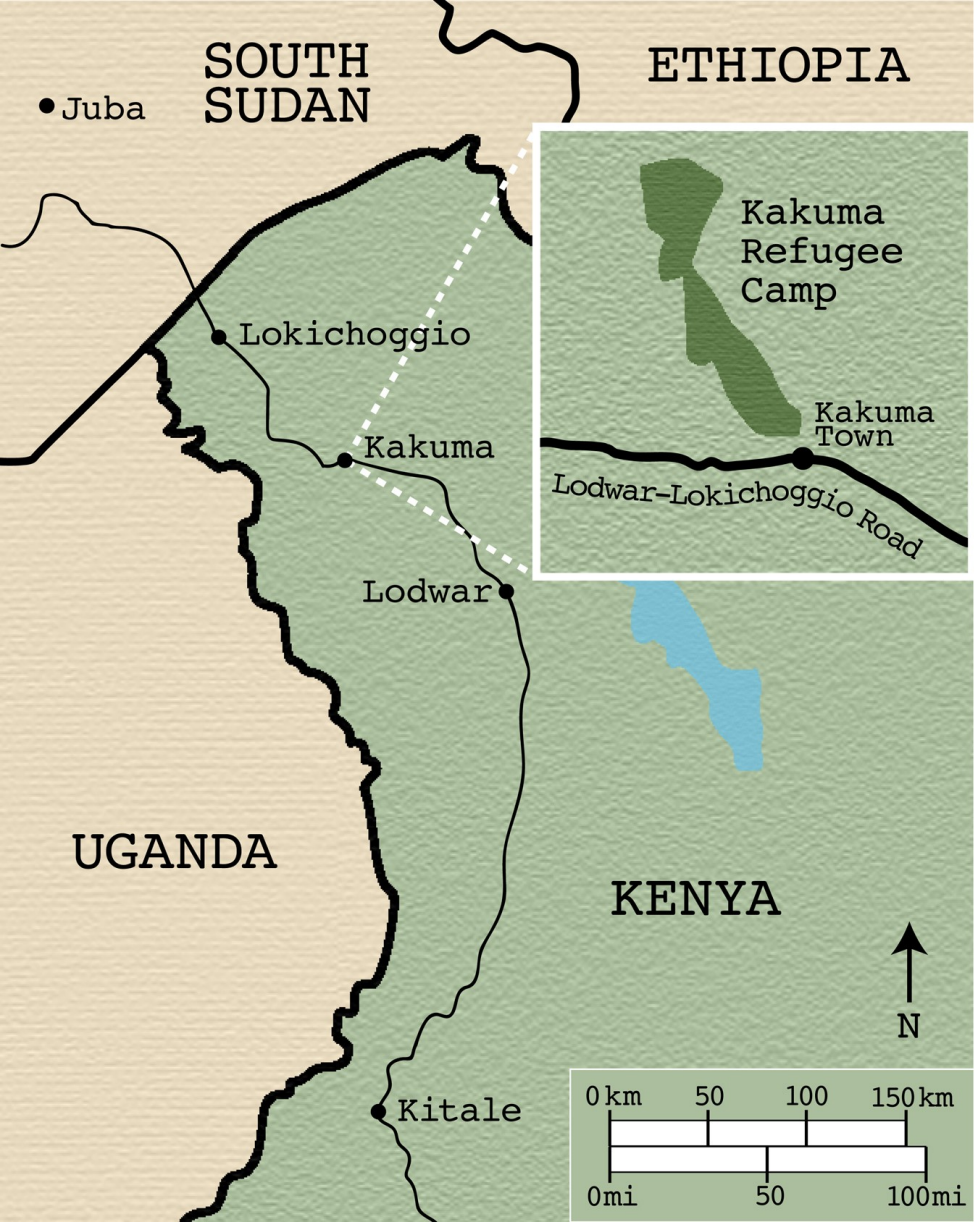
Map of Kakuma Town and Kakuma Refugee Camp. Courtesy of Rahul Oka.

Kakuma Refugee Camp, located near the of South Sudan border in northwestern Kenya, is one of the largest refugee camps in the world (Fig 1). Established in 1992, the camp has grown considerably from a population of 85,000 in 2011 to more than 160,000 in September 2016, due to the crises in Somalia and South Sudan [11]. The camp itself is managed by the UNHCR and the Camp Manager’s Office under the Refugee Affairs Secretariat of the Government of Kenya, whereas the camp’s healthcare services are managed by the International Rescue Committee. The Kakuma camp is divided into 4 sections (Kakuma 1, Kakuma 2, etc.) and, as of 2016, is associated with Kalobeyei—an extension of the camp intended to eventually be an integrated settlement comprising 3 villages (Kalobeyei 1, Kalobeyei 2, etc.). The larger sections/villages are further split up into numbered zones, and then again further into numbered blocks [12].

There are unique attributes of the camp that contribute to the political economy of insecurity. The camp hosts refugees from various different countries of origin—Sudan, 5.5%; South Sudan, 55.6%; Somalia, 21.3%; Ethiopia, 5.7%; Uganda, 0.8%; Rwanda, 0.3%; Burundi, 4.7%; and Democratic Republic of the Congo, 5.9% [13]. The various ethnic groups’ having varying levels of resources fosters an insecure environment for women through increasing risk of both inter-ethnic/inter-clan conflict and domestic conflict [6]. Additionally, under Kenyan law, refugees are prohibited from keeping livestock or seeking employment outside the camp and are left to rely on rations that are often culturally inappropriate, consisting of food products that are not consistent with the regular cultural diet of various refugee groups [14]. As a consequence, refugees have created an informal economy of various stores and restaurants within the camp. Previous research has shown that Somali and Ethiopian Oromo families have control of this economy, and therefore tend to have access to more resources than other ethnic groups in the camp [14].

Both camp residents and authorities have confirmed chronically high levels of insecurity in Kakuma Refugee Camp for the past couple of decades [5]. Recent reviews of insecurity at the camp revealed many women felt limited support and security from their community, police authorities, and humanitarian workers [6]. As the camp has continued to expand due to the increase in refugees fleeing conflict, there are various parts of the camp that are less established and less secure. For example, in Kakuma 1 and 2 (the oldest parts of the camp), over time, refugees have been building gates and locking doors to increase the security of their families’ claimed spaces. The newest part of the camp, Kalobeyei, however, is 3.5 km from the Kakuma camp and is not as developed, with tents serving as housing. Therefore, Kakuma Refugee Camp serves as an ideal case study to examine the various sources insecurity for refugee women in an encamped refugee setting.

The purpose of this qualitative study is to better understand what refugee women experience while living in refugee settings. This study specifically explores the sources of insecurity and personal experiences of Ethiopian Oromo and Somali women while residing in Kakuma Refugee Camp.

## Methods

This study was part of an overall larger ethnographic study in Kakuma Refugee Camp. Rahul Oka is an anthropologist at the University of Notre Dame and has conducted ethnographic and survey research in Kakuma Refugee Camp and the northwestern Turkana area from June 2008 to the present. During this time, his team, consisting of graduate students from the University of Notre Dame and local research associates from refugee and host communities, has developed social relationships based on trust and reciprocity with members of both the various refugee ethnic groups (Somali, Oromo, Dinka, Nuer, Rwandan, Burundian, Congolese) and the Turkana host communities. These relationships have enabled the team to collect data on covert and overt socioeconomic behaviors and trade/exchange networks, black markets, informal/extra-legal activities, use of relief products, psychosocial stressors, biomarkers of stress and nutrition, narratives and observations of conflict and coexistence, and factors underpinning the resilience and robustness of social economies within and between the refugee and host communities.

This particular study, focusing on the insecurity of women within the camp, utilized an ethnographic approach focusing on semi-structured interviews [15]. This study is reported as per the Consolidated Criteria for Reporting Qualitative Research (COREQ) guideline (S1 Checklist). The research team consisted of a graduate student (now MS), a professor in anthropology (PhD), an adjunct professor in research design and qualitative methods (PhD), a former graduate student and current lawyer (MS, JD) and research assistants within the refugee community. Between May 23 and June 30, 2017, 20 women were recruited to participate in ethnographic semi-structured qualitative interviews. The interview guide was structured based on general perceptions of security, sources of insecurity, and experiences during pregnancy. Interviews included both open-ended questions and specific questions focused on experiences and perceptions of insecurity walking around the camp and within the women’s own homes, overall conditions of the camp, locations that were more dangerous, and overall experiences while pregnant.

### Sampling and recruitment

The population of the study was limited to Somali and Oromo women 18 years and older (with no upper age limit) who had given birth at least once in Kakuma Refugee Camp. Previous work by Oka and colleagues suggested that the Somali network (and, to some extent, that of the Oromo) dominates the local informal economy and tends to have better access to more resources than other ethnic groups in the camp [11,14]. To better control for variation in different experiences based on economic status, we sought to isolate insecurity related to the refugee experience, and not economic status, which is why we focused on this prosperous group within the Kakuma Refugee Camp.

Due to both the infeasibility of collecting a random sample within the camp and the sensitivity of the topic, interview participants were selected through a snowball sampling approach through contacts used in previous research, including a hired mobilizer with previous experience in similar research. The mobilizer was responsible for recruiting women fitting the criteria outlined above who were willing to talk about their pregnancy experiences and stressors [11]. Women living in Kalobeyei, the newer and less developed camp, were also included in this study at the suggestion of the mobilizer, who believed that these women had more issues with insecurity than those living in Kakuma 1, an older and more established camp section. No prior relationship existed between the interviewer and the prospective participants; however, the mobilizer and translator naturally often had relationships with participants due to the nature of the tightknit community within the camp.

### Interviews

The interview guide was constructed to gain a better understanding of the mothers’ experiences within the camp. Previous qualitative studies concerning security and violence in Kakuma Refugee Camp [6,7] were used to guide the composition of specific interview questions. While this paper only focuses on experiences of insecurity, the interview guide was originally structured to encompass a broader range of experiences of living in the camp and was based on the following themes: (1) general worries/feelings of stress, (2) general perceptions of insecurity including experiences in the house and experiences in the camp, (3) general perception of own health, and (4) experiences during pregnancies—including complications, caretaking, and access to services (S1 Text). The researcher (AL) reviewed the interview guides with the hired translator and research assistants (2 Oromo women, 1 Oromo man, and 2 Somali women) to ensure it was culturally and linguistically appropriate for both Oromo and Somali women.

The interviews lasted 30–60 minutes each. They were conducted in a place of each participant’s choosing, typically her own home, with the assistance of a translator. The interviews were all carried out by the same female researcher (AL) and the same female translator (AS). The researcher was a trained interviewer with previous interview experience in rural settings and was enrolled in the Master of Science in Global Health Program at the University of Notre Dame during the time of the study. The translator was a refugee of Ethiopian Oromo descent living in Kakuma Refugee Camp herself. She was one of the few Ethiopian women within the camp to finish all of primary schooling and was an experienced translator fluent in English, Oromo, Somali, and Swahili. She was identified by a trusted research assistant whom the research group had worked with for several years. The interviewer conducted a 2-day training for the translator, which included practice interview sessions. Children were usually present during the interviews, and at times men were also present during the interviews. The researcher asked the questions in English, and the translator translated the questions as close to verbatim as possible for the participant. Due to institutional review board constraints, and in order to maintain rapport with the participants, interviews were not recorded; however, detailed and verbatim notes of the translated responses were taken by the researcher during the interview and typed up within 24 hours. Participants were informed that the researchers involved in the study were from a university and were not affiliated with UNHCR or other services within the camp, and that they could not provide services beyond listening and disseminating the participants’ stories in their research. The participants acknowledged their understanding that the researchers were interested in qualitative research as it pertained to their experiences as women within the camp. Each participant gave oral informed consent; written consent forms were deemed to be intimidating and overly formal by research assistants and previous researchers who had worked with this community. No women declined to participate.

Interviews were conducted until data saturation was achieved. Twenty interviews were conducted in total. After the initial interviews, 12 of the 20 interviews were identified as requiring further clarification. These 12 interviews were repeated with the corresponding interviewee to cross-verify that the relevant meaning had been captured and to expand details within the respondents’ interviews. At the end of the first interview, each woman was given 500 Kenyan shillings (US$4.97/£3.84/€4.48). Women who were interviewed twice also received a small bangle or pair of earrings, in anticipation of Eid al-Fitr. To preserve anonymity, each participant us identified by an interview number (Interview 1, Interview 2, etc.).

### Research observation

As pure participant observation could not be obtained in a refugee setting, a mix of participant and research observation through events such as food sharing was used to supplement interviews, in accordance with Oka and colleagues’ previous anthropological work in Kakuma Refugee Camp [11,14]. Observation was done during evenings and on days that interviews were not scheduled. Field notes from observation were written up after the events and were not included in the interview transcripts. Notes also included thoughts and interpretations of the research assistants that were shared during both casual conversations and informal post-interview debriefing sessions utilizing principles of reflexivity.

### Analysis

Using research observation and notes to contextualize data, the head researcher (AL) developed a codebook utilizing both an inductive and deductive approach. Typed and de-identified interview notes were uploaded to Dedoose, a qualitative analysis program, and given codes and subcodes from the aforementioned codebook. Codes included insecurity, health, pregnancy experiences, healthcare facilities, stressors, income, coping, and support. The subcodes of insecurity included general insecurity, host community, refugee community, healthcare facilities, issues regarding security, and suggestions for improvement. Each subcode was summarized in the form of a short paragraph. All authors participated in the final analysis, during which some subcodes were grouped together into overall themes related to the research question. Notes from observation were used to guide the research focus, further interpret stories, and identify several patterns in interview themes. Themes were organized into a cohesive narrative. Key quotes from the translator’s interpretations of participant responses were identified to provide further evidence for each theme. Few verbatim quotes were obtained; therefore, the stories told by the interviewees are reported and conveyed through the translator’s paraphrased words. True verbatim translated quotes are indicated by quotation marks.

### Ethics statement

These methods were approved by the University of Notre Dame Institutional Review Board (protocol ID: 15-05-2534) in May 2017. Oral consent was provided by all participants in this study.

## Results

Interviews explored sources of insecurity and experiences in pregnancy by location, timing, and special circumstances such as seeking services (e.g., healthcare, rations). Demographics of participants are described in Table 1.

**Table 1:** Demographics of participants.

Characteristic	Number (%)* (*n* = 20)
Age range	19–40 years
Ethnic group	
Somali	10 (50%)
Ethiopian (Oromo**)	10 (50%)
Residence	
Kalobeyei	6 (30%)
Kakuma	14 (70%)
Single/widowed/divorced	6 (30%)
No formal education	12 (60%)

*Unless specified otherwise.

**One woman who belonged to another ethnic group but who married into an Oromo family was included in the Oromo group due to her ties with the Oromo network.

Overall, the interviews wove together a common narrative showing that women feel there is no safe place for them. Women felt various levels of unavoidable insecurity due to (1) tensions with the host community, (2) intra- or intercultural conflict within the refugee community, (3) issues with camp security personnel, and (4) unsafe situations in accessing healthcare.

### Tensions between host community and refugee community

Tension between the host community and refugee community is at the root of most insecurity within private spaces—defined as home and within one’s block. Within the interviews, insecurity, particularly at night, was indicated as a chronic stressor for all women. A few women even stated that Kakuma camp was less safe than their previous situation (home country or Dadaab Refugee Camp). Women prominently talked about insecurity while they were in their homes at night due to thieves who rob, rape, and kill people.

I don’t feel safe within the camp because I’m a single mother and within this camp every night, there are thieves from Turkana who attack people. They kill, rob, and rape women, which I can be openly a victim of that act because I’m living alone. (Interview 15)

Most women indicated that they repeatedly heard gunshots outside at night; many indicated the gun shots as the main source of their constant fear.

At night, gun shots are there and they kill people. Ladies need a man for support. I am alone and when I hear [the gun shots] I feel like I’m going to die. (Interview 3)

While there was some confusion as to who the thieves were, most women stated they believed them to be Turkana (the host community) individuals coming into the camp when women were in their homes for the night. The perception of the thieves being from the host community has continued to foster mistrust between the refugee community and the host community.

The host community are the ones who rape the girls. They don’t even spare the girls and women. They rape all. (Interview 7)

In both researcher observation and interviews, women indicated that while there was a set curfew (6:00 PM), it was useless because there was nothing halting the host community’s entrance after 6:00 PM. Women, including research assistants, indicated they did not leave their own home and/or zone after curfew because it was too dangerous.

No woman stated she felt safe from attacks at night, regardless of her housing situation; however, some women revealed some places were less safe than others. In fact, a few women stated they moved homes to gain a little more security after a nearby attack. One Somali woman, in particular, moved from a Somali block into an Oromo block because she was attacked (Interview 6).

My biggest problem in Kakuma is about insecurity. Before I did not live here. I used to live in block 4 and [a] group of thieves broke into our house. They took everything we have and they beat my mom. That’s why I had to move to this house. (Interview 6)

She believed the thieves targeted the Somali blocks more often for 2 reasons: (1) Somalis are known to have more money through their business ties and (2) the Somali block is now less densely populated due to a recent wave of repatriation.

In contrast to women of Kakuma 1, women in Kalobeyei felt their living situations were less secure due to the material of their houses and the lack of the secure gated systems that Kakuma 1 has developed over time. The houses in Kalobeyei are made of tent-like plastic walls provided by UNHCR, which can easily be accessed and ripped, making crime and theft more accessible. In Kakuma 1, refugees have implemented their own “street lights” for the night, and the houses are made of large iron sheets or a combination of wood and mud, making it more difficult for someone to easily access the house. However, a few of the women pointed out an even more dangerous area in Kalobeyei, near the river, where attacks frequently occur because the thieves can escape easily into the river.

Since I came here, I have just been through fear. … These houses are made of plastics. It can be torn any time. … I do exist in fear every now and then. [Interviewer: When did you feel the most scared?] The day we were attacked by a gang of thieves who were Turkana. They attacked our neighbor. They started shooting at people so that they scare them away. (Interview 14)

I have a neighbor who was attacked by gangsters 2–3 times and they had to tear the plastics in order to get into the house, they started screaming. There was not anyone who went to help. If my neighbor is being attacked and they are screaming, how about me? How will I feel safe? I might be the next victim. I don’t even get sleep at night. I’m just waiting for my day. (Interview 11)

In particular, some women in Kakuma 1 pointed to a specific time in 2016 as a time of maximum fear. The camp was attacked regularly in sequential nightly attacks. Since then, women believed the occurrence of crime had decreased, but the fear still remained, and many women often wondered when their time of victimization would come.

Last year, when they raped in Kakuma 4 and in Kakuma 1, four girls who were under 18 and they killed their brothers. That’s when I get scared because I fear I might be next. … Because I hear gunshots every night and I have witnessed girls who have been raped [not in the act but in recalling]. … In 2016 [over the course of one month], every night, they were raping girls. (Interview 7)

Mothers with daughters expressed their fear for their daughters’ security, even above their own. Many women confirmed that women were less safe in the camp because they are more vulnerable to attack through kidnap and rape.

The way of life is very hard. I have four daughters and three boys and anyone staying in the camp while having daughters is not safe because it is not secure for girls because they rape girls at night and they kidnap during the day. That’s why it is not safe for me. (Interview 7)

In 2016, last year, when thieves were everywhere, we had to send our daughters away to Nairobi to some people for help so that they can secure our daughters. Just for a while. … There was a day that a young girl was shot. I got scared because I also have daughters at home. … I was fearing. This year nothing has happened, we are all okay. (Interview 17)

### Intra- and intercultural conflict within refugee community

Tension within the refugee community itself extends across both private and public spaces. While “host community” thieves and robbers in the night were the main source of insecurity issues for refugees in the camp, perceptions of insecurity within the community also seemed to be a chronic stressor.

People fear host communities, but I also fear my community members because they might seek revenge through my daughters to harm them in different ways. (Interview 4)

However, unlike fear of host communities, perceptions of insecurity within the refugee community had many variations in the source: from rival tribes, from within one’s own tribe, and from within one’s own family. One woman mentioned abuse from individuals of her own nationality (Ethiopian) due to her minority tribe (Interview 4). It got to the point where she felt that she couldn’t even go to the community water tap without being harassed.

If I am faced with many problems, I cannot get help. Needs like fetching water has become a problem for me because going to the tap is not safe. … They abuse me. They tell me, ‘This one is [ethnic group].’ Sometimes, they go to an extent where they threaten me and try to do some harm. … My children are suffering because of me. They can’t mix with the people because I feel unsafe and my husband feels unsafe. My husband has to go to the school 3–4 times to ensure that the children are safe. (Interview 4)

Another woman stated that a community member who supposedly had a mental disorder was harassing her. He would throw stones at her every day while she was pregnant. He harassed her so much that she had to move to an abandoned house in order to seek refuge. Another woman mentioned feeling slightly harassed and not supported by the community because she had a child out of wedlock (Interview 20). And another 2 women stated they were receiving severe threats from their ex-husband and/or ex-husband’s family to abduct their children.

After my husband left me, I have an elder daughter whom I have taken to boarding school. But after he left me, he brought up another problem. In 2016, when my daughters were in school, there was a man who lived in Nairobi and they agreed upon giving out my daughter to him in marriage because he wanted money. He [my ex-husband], came back to me then he told me that he’s going to take me for treatment and my son was sick and said he’d treat him also, the son. So for this, we should give out our daughter because I need money. That’s when I told him I’m not someone that is raised by his parents. I grew up in another woman’s home, while washing utensils and clothes without getting educated. I don’t want my daughter’s future to be like that of mine. That’s when he threatened to kill me and when my daughter heard about it she had to run away to the protection office and police. (Interview 9)

Interestingly, women tended to tell these stories of family issues in more detail than the stories of robberies and attacks during the night, especially if the issue was affecting their children.

### Camp security issues

Camp staff and security personnel contribute in 2 ways to feelings of insecurity within public spaces, by direct harmful actions towards refugees and by the lack of action taken for refugee complaints. The field house and food ration distribution center were both identified as well-known places where refugees, including women, receive such treatment. Some women mentioned seeing or experiencing a beating from a security guard/staff member.

I feel insecure within the camp and even the policemen, instead of securing people, they are the ones who sometimes rape and even kill. When you go to complain, they ask for bribes or else your case is forgotten. No one talks about it. (Interview 7)

Life in Kakuma is such a life that a person as a refugee does not face any thing other than difficulties. … We have to go too far distances and [at] the ration center, people are too many and the line is very long for one to access the ration place easily. When we are trying to access the food center, we have the host staff who beat people and they don’t differentiate if that person is a lady or a man, a pregnant woman, or a nursing woman. They just beat all of them. (Interview 18)

Women also cited direct impacts of insecurity on pregnancy, as a few women openly spoke about 3 instances of seeing (or experiencing) a pregnant woman being beaten by a security guard, staff member, or policeman at the ration center or food distribution center. In all of the accounts of these instances, the pregnancy ended in a miscarriage, still birth, or neonatal death.

I went to the ration place and I was beaten there by the policeman. Then I started bleeding and I was taken to the hospital, I lost a lot of blood. That’s when I have birth to premature baby. The baby was given oxygen and was put in an incubator. He stayed for two days and he died. He could not take even the breast. (Interview 4)

While not all women brought up direct physical abuse by the security team and staff, many spoke of lack of action by the security team and staff and felt that there were no services willing to aid them when they felt unsafe from their own camp community. Some women stated there was no way to report anything and have a prompt response. For Kalobeyei, there is no place close to report any security problems as both the UNHCR offices and the police station were located closer to the main camp in Kakuma 1. It was also difficult to access the offices, especially without being asked for money.

Because they have made me believe, the ones who have money are the ones who survive. Whenever I go to the UNHCR, I’m not allowed to access the offices. In order to access the field post, the security guards need money to let you in, but I don’t have any. (Interview 4)

A few women also stated that the security team was of no help because they were unarmed and they also feared the armed thieves. Sometimes they came when everything was over because they also feared for their lives.

### Access to healthcare

Women potentially endanger themselves and their unborn children through the dangers of traveling to clinics for care and from receiving poor/biased treatment in health clinics. Additionally, women often felt less safe during pregnancy. When asked, many women stated that the amount of stressors was equal while pregnant, but some women stated they felt less safe due to the extra precautions they had to take as a pregnant woman since they were caring for not only themselves, but also their baby.

Pregnancy can also put women in insecure situations they might not typically find themselves in, sometimes stemming from pressure to deliver in the clinic. After calling the ambulance while in labor, some women in Kalobeyei 2 disclosed that they were forced to walk long distances along the highway in order to access the ambulance. Some women stated they were forced to walk late at night for 4 kilometers, taking approximately 2 hours, to gain access to the ambulance and still had to wait for it to arrive, showcasing the difficulty for women in accessing health facilities safely during an emergency.

When I was caught with labor pain, I had to walk to the hospital during the night because we did not have any ambulance. He refused to come. I walked to Red Cross health post by highway at 2 AM. (Interview 14)

Being a refugee is very hard and after all being a woman, it being a difficult life is something obvious. When I was about to give birth, I did not get any ambulance. No ambulance comes here to get you unless you go to the highway and when we get to the high way, we have to wait for some hours for them to come. (Interview 13)

After delivering, one of the participants also reported being dropped off near the highway to walk back to her home with her newly delivered baby.

When he was bringing us back, he made us get out on the highway and then we tried to tell them to take us home, he told us that he is not supposed to take everyone to their home. … So that’s a problem. They made me walk from the highway to my house with a newborn baby. (Interview 13)

Even with a male friend as an escort, walking alone a night as a female, especially in labor, was noted as a severe risk for violence (from the host community), especially when unarmed.

Additionally, when asked about their experience with health and pregnancy care, interviewed women also indicated the healthcare services as an additional—but not main—source of stress and, at times, insecurity. Many women described their healthcare experiences as negative and, in some instances, traumatizing. Furthermore, women talked about the lack of respect and lack of dignified care they received. Reports of lack of respect ranged in severity. Some women indicated they were treated like animals and inhumanely.

Whenever we go to the hospital, they just call us with names “Mama Kalobeyei.” So they have branded that name and they don’t even treat us like human beings. … There is not even respect when you go there and the first sight when they see you, then how will you expect that you will deliver safely? (Interview 19)

In more extreme examples of insecurity at the time of delivery, many women described their own traumatic experience or one that they witnessed.

They ill-treat you. They disrespect people. When you tell them to come and help you during the pain, they tell you not to disturb them. They even beat a girl, after beating her, the baby came down on the floor. … She was told to go away and walk around in the hospital during her labor pains. (Interview 13)

“They took me into the delivery room at 8:00 PM with no one around me. They made me spend the whole night and I got out of that room at 7:00 AM in the morning. Just like a slaughtered goat, blood was flowing from me and they tore my clothes on me. After then, I could not recognize myself.” (Interview 2)

“A woman went to them for check up because she was feeling pain then they told her the delivery is not yet. Then after, she went to the toilet. She gave birth near the toilet.” (Interview 2)

Many women indicated they had been asked for money in order to obtain proper and respectful care, further illustrating how lack of income creates disparity for women.

In the hospital we are not given good medicine and sometimes they deny to give you [prescriptions], whenever we take a woman who is going to deliver, they usually ask for bribes, minimum of 3000 KSH [Kenyan shillings]. They have denied me the [prescription paper] for asthma. A woman’s respect and dignity is not considered. (Interview 17)

They [nurses and refugee nursing assistants] asked me, ‘Are you Ethiopian or Somali?’ then I had to give them ‘kitu kidogo’ [translated from Swahili to ‘a little something’]. (Interview 2)

This is especially concerning for women in labor who are experiencing complications. One woman stated that the hospital staff would tell you that you had complications so that you were scared and would pay the staff extra to receive proper care (Interview 2). Another woman stated patients were only respected if a member of their own tribe treated them—then they received good treatment (Interview 5). Interestingly, when interview participants were asked why women sought delivery at the healthcare facilities, even after reporting negative experiences, the main responses were that it was necessary to get the birth certificate or that they lacked help within the community to deliver safely. A few women indicated they would feel safer delivering at home but feared for their child’s chance of receiving a Kenyan birth certificate. While UNHCR confirmed to the authors that this is not true, it remains a common misconception that one must deliver in a health facility to receive a valid birth certificate.

Honestly, it is better I deliver at home, but there is just one thing in the way. If I don’t deliver at the hospital, I don’t get the birth certificate. (Interview 2)

However, not all women showcased the same frustration with the health facilities. Some Somali women described their healthcare experience in a positive light, stating that while it was not perfect, the healthcare staff did provide adequate care.

When asked what their ideal experience with the hospital would be, most women stated that they wished for a facility where they could be treated with dignity and respect.

It would have made me feel safe if the clinical staff treated me in a good manner, but that doesn’t happen. … I mean that we should be treated well, respected, given the right treatment. We should be taken care of. We should not be thrown like a dog. They should give us morale instead of disrespect. (Interview 2)

## Discussion

The results from our study show that the sources of insecurity, even for women from the ethnic groups with the most connections to the informal economy of Kakuma Refugee Camp, stem from tensions with the host community, intra- or intercultural conflict within the refugee community, mistrust of security personnel and camp staff, and unsafe situations in accessing care, including obstetric violence. Taken together, there is no truly secure place for women within the camp.

There are many underlying issues that contribute to this insecurity. Many of these problems stem from the lack of power that refugees have in their own lives, as has been shown in previous studies cited below. Fundamentally, refugees are forced into a new country and new environment occupied by a preexisting low-resource community. In the case of our study, refugees recalled that the host community’s frustration in not receiving services (food, healthcare, etc.) that they had been promised by various organizations led to threats that they would continue to attack the refugee camp until they received the aid that they were promised.

Previous literature has documented that tension between the host community and refugee community is not novel to refugee camps [16–19]. In our study, we further found that women living in Kalobeyei—far away from the main camp—felt they were at higher risk for violent acts by the host community due to lack of secure environments and established housing. However, it is also important to note that much of the literature regarding insecurity issues between refugee and host communities focuses on the insecurity that the refugee community brings to host countries rather than the insecurity the host community brings to the refugee community [20,21]. This disparity in literature on the host versus refugee point of view in all likelihood contributes to the overall negative attitudes towards refugee communities and continues to act as a catalyst in creating tensions that lead to insecurity for both parties.

Further, refugee women revealed lack of trust towards security personnel and camp staff due to direct abuse and neglect when reporting incidents. This information is critical in mitigating conflict both with the host community and within the refugee community. Previous research has reported violence accompanying militarized policing within Kakuma Refugee Camp, including physical force and bribery, similar to the findings in this paper [22]. Another study in Kakuma Refugee Camp that focused specifically on women revealed difficulties in reporting incidents, especially in reporting SGBV, due to the tedious and lengthy process [7]. While intimate partner violence did not come up in our interviews, a previous study in Kakuma Refugee Camp reported other logistical challenges in reporting such violence, including fear of delaying the resettlement process [23]. However, issues with security are not isolated to Kakuma Refugee Camp; they extend to other camps globally. A review of security and SGBV in a refugee camp in Tanzania reported similar barriers to safety for women [24]. This analysis offered possible solutions, including more inclusive large-scale decision-making such as by incorporating refugee guards into the security system and consulting refugee women in the layout of the camp [24].

Lastly, refugee women did not feel safe when seeking healthcare. Women living in Kalobeyei described increased difficulty in accessing healthcare due to their distance from healthcare facilities and lack of safe transportation. Mistreatment within healthcare settings themselves has likewise been well documented in similar settings. Previous studies have revealed that women in Kenya often endure different forms of mistreatment, including verbal abuse, physical abuse, neglect, discrimination, abandonment, poor rapport, and failure of the health system to uphold professional standards upon childbirth [25,26]. While there is no current public research concerning obstetric violence in refugee camps/settings, there are media depicting this issue in refugee settings in Greece [27].

Overall, even the most “privileged” refugees may be extensively negatively affected by societal tensions and cultural norms, because they have no voice or power in their current environment. Our study has established that the Oromo women who lived in Kalobeyei with less physical access to the commercial market and services felt less secure than those at the main camp. If fear of violence and insecurity is so prevalent among women of relative means, such as the women in this study, we can presume that women of other ethnic groups in the camp, who do not have the same access to the community economy, may experience even more violence than the Oromo or Somali women.

It is important to note that this study only reports the voices and opinions of refugee women of Somali and Oromo descent and does not contain opinions from camp staff/security personnel, UNCHR staff, healthcare facility staff, or other decision-makers regarding Kakuma Refugee Camp. Limitations of this study include possible selection bias due to the snowball sampling; however, this was the only feasible methodology within this setting. We opted against randomization for cultural reasons and based on evidence that previous attempts to impose random household visits on refugees have usually resulted in imprecise and inaccurate responses, usually due to survey fatigue [11,19]. Additionally, snowball sampling allowed us to identify women who were willing to tell personal stories in a safe environment. The sample was also limited to Somali and Ethiopian Oromo women, and thus their experiences cannot be generalized to other ethnic groups within the camp. Other limitations include inability to record the interviews and the presence of men/children in various interviews, which may have impacted how women described their experiences. Lastly, many of the quotes are not verbatim and may be subject to subtle yet important inaccuracies in translation. To mitigate this, we utilized a translator who was familiar with the local languages and finer nuances, and the interviews were often done twice to verify understanding of previous interviews. ​While using a community member as a translator may have also impacted how women responded in ​interviews due to previously established relationships, we believe it was to our advantage as it provided trust between the research team and interviewees.

Given that insecurity has been linked to increased stress and adverse health outcomes, this study has many implications about the well-being of women in refugee camps similar to Kakuma Refugee Camp. Studies have shown that exposure to violence and increased stress is associated with an increase in gynecological conditions, adverse physical and mental health outcomes [10,9,28], and adverse pregnancy outcomes including low-birth-weight babies and obstetric complications [29–31]. Many of these studies consider adverse health outcomes to be mediated by increased stress [28–30]. Previous work has revealed that women who are able to access the commercial markets are able to cope with their fear and stress through increased meal sharing with culturally familiar foods, often enriched with processed sugars and oils, which may also indirectly contribute to women’s health [32].

Positive health outcomes are dependent on a multitude of factors. This study has shown that there are barriers for women in refugee camps in accessing critical health services. In order to improve health outcomes and overall well-being for women living in refugee camps like Kakuma Refugee Camp, policies must first be put in place to increase safe spaces for women, especially women in newer areas of the camp who may have less structural security. Policies need to be multifaceted not only to increase healthcare access but to disrupt the sources of the insecurities these women continue to face.

Interventions in the public sphere should be aimed at public security efforts, including protecting refugees from corruption, public shaming, and beatings. Previous successful interventions, as the literature cited above has shown, have included giving autonomy back to the refugees when it comes to large-scale decision-making regarding their own security, and employing refugees as security guards [24]. Interventions at the clinical level need to begin with staff cultural sensitivity training that includes recognizing signs of the social determinants that are reducing the overall well-being of women in refugee camps. On a more procedural level, there is a need for a more streamlined protocol for refugees accessing emergency care by ambulance that ensures their safety before arriving and after leaving the hospital.

By implementing a cross-sectional, collaborative policy that addresses tensions that arise with the host community, intra- and intercultural conflict within the refugee community, and unsafe situations in accessing healthcare–including obstetric violence, we can reduce the stressors found in the everyday life of refugee women and ultimately reduce the insecurity and instability felt by women. As the number of refugees is expected to continue to increase worldwide, it is crucial that we work to protect this vulnerable population within a vulnerable setting. By creating a safer environment for women in both private and public areas found in refugee camps, we can work to mitigate the social determinants of health and improve the overall health and well-being of this particularly vulnerable population.

Future research should aim to explore similarities and differences in insecurity for women of other ethnic groups residing in Kakuma Refugee Camp as well as for refugee women in other camps. There are also many camps in which refugees are integrated into the host community economy. Further research should look into insecurity within these integrated camps to explore if integration mitigates host–refugee tension and violence.

Overall, this study suggests that women living in refugee camps often exchange one state of insecurity in their home country for another in their refugee camp. Refugee settings may create this new form of insecurity for women at home, in public areas, and even in healthcare settings. This is both a human rights issue and a key public health issue, as insecure settings are linked to poor health outcomes, especially during pregnancy. It is imperative that both medical and nonmedical professionals working in these settings understand the sources of insecurity faced by women in refugee camps and use this information to guide projects/initiatives for improving security services and access to healthcare in displaced settings. By creating safer environments for women in refugee camps, we can improve health outcomes for them and for their babies.

## Supporting information

S1 Checklist (DOCX)

S1 Text Interview guide. (DOCX)

## Abbreviations

SGBV: sexual and gender-based violence
UNHCR: United Nations High Commissioner for Refugees
