# Supplementary material for: Exploring sources of insecurity for Ethiopian Oromo and Somali women who have given birth in Kakuma Refugee Camp: A Qualitative Study
Source: PLoS Med. 2020 Mar 24;17(3):e1003066. doi: 10.1371/journal.pmed.1003066 (PMC7092956; doi:10.1371/journal.pmed.1003066)
Supplement: S1 Checklist — (DOCX) [file pmed.1003066.s001.docx]

### **Consolidated criteria for reporting qualitative studies (COREQ): 32-item checklist**

| **No** | **Item** | **Guide questions/description** |
| --- | --- | --- |
| **Domain 1: Research team and reflexivity** | | |
| Personal Characteristics | | |
| 1. | Interviewer/facilitator | The interviews were all carried out by the same female researcher (AL) and the same female translator. [Methods Paragraph 6] |
| 2. | Credentials | The research team consisted of a graduate student (now MS), a professor in Anthropology (PhD), an adjunct professor in research design and qualitative methods (PhD), a former graduate student and current Lawyer (MS, JD), and research assistants within the refugee community. [Methods Paragraph 2] |
| 3. | Occupation | The research team consisted of a graduate student (now MS), a professor in Anthropology (PhD), an adjunct professor in research design and qualitative methods (PhD), a former graduate student and current Lawyer (MS, JD), and research assistants within the refugee community. [Methods Paragraph 2] |
| 4. | Gender | The interviews were all carried out by the same female researcher (AL) and the same female translator. [Methods Paragraph 6] |
| 5. | Experience and training | The researcher was a trained interviewer with previous interview experience in rural settings and was enrolled in the Master of Science in Global Health Program at the University of Notre Dame during the time of study. [Methods Paragraph 6] |
| Relationship with participants | | |
| 6. | Relationship established | No prior relationship existed between the interviewer and the prospective participants; however, the mobilizer and translator naturally often had relationships to participants due to the nature of the tightknit community within the camp. [Methods Paragraph 4] |
| 7. | Participant knowledge of the interviewer | Participants were informed that the researchers were from a university and were not affiliated with the UNHCR or other services within the camp and could not services beyond their research. [Methods Paragraph 6] |
| 8. | Interviewer characteristics | The participants acknowledged their understanding that the researchers were interested in qualitative research as it pertained to their experiences as women within the camp. [Methods Paragraph 6] |
| **Domain 2: Study design** | | |
| Theoretical framework | | |
| 9. | Methodological orientation and Theory | This particular study, focusing on the insecurity of women within the camp, utilized an ethnographic approach focusing on semi-structured interviews. [Methods Paragraph 2] |
| Participant selection | | |
| 10. | Sampling | Due to both the infeasibility of collecting a random sample within the camp and the sensitivity of the topic, participants of interviews were selected through a snowball sampling approach through contacts used in previous research, including a hired mobilizer with previous experience in similar research. [Methods Paragraph 4] |
| 11. | Method of approach | The mobilizer was responsible for recruiting women fitting the criteria outlined above who were willing to talk about their pregnancy experiences and stressors... [Methods Paragraph 4] |
| 12. | Sample size | Twenty interviews were conducted in total. [Methods Paragraph 7] |
| 13. | Non-participation | No women declined to participate. [Methods Paragraph 6] |
| Setting | | |
| 14. | Setting of data collection | They were conducted in a place of each participant’s choosing, typically their own homes, with the assistance of a translator. [Methods Paragraph 6] |
| 15. | Presence of non-participants | Children were usually present during the interviews and at times, men were also present during the interview. [Methods Paragraph 6] |
| 16. | Description of sample | See Table 1. [Results] |
| Data collection | | |
| 17. | Interview guide | The interview guide was constructed to gain a better understanding of the mothers’ experiences within the camp. Previous qualitative studies concerning security and violence in Kakuma [6,7]were used to guide the composition of specific interview questions. While this paper only focuses on the insecurity women experienced, the interview guide was originally structured to encompass a broader range of experiences of living in the camp and was based on the following themes: 1) General worries/feelings of stress, 2) general perceptions of insecurity including experiences in the house, experiences in the camp, 3) general perception of own health, and 4) experiences during pregnancies- including complications, care taking, and access to services (S1 Text). [Methods Paragraph 2] |
| 18. | Repeat interviews | After the initial interviews, twelve of the twenty interviews were identified that required further clarification. These twelve interviews were repeated with the corresponding interviewee to cross-verify that the relevant meaning had been captured and to expand details within the respondents’ interviews. [Methods Paragraph 7] |
| 19. | Audio/visual recording | Due to IRB constraints and in order to maintain rapport with the participants, interviews were not recorded; however, detailed and verbatim notes from the translator were taken during the interview and typed up within twenty-four hours. [Methods Paragraph 6] |
| 20. | Field notes | As pure participant observation could not be obtained in a refugee setting, a mix of participant and research observation through events such as food sharing was used to supplement interviews, in accordance with Dr. Rahul Oka’s previous anthropological work in Kakuma [11, 13]. Observation was done during evenings and on days that interviews were not scheduled. Field notes from observation were written up after the events and were not included in the interview transcripts. Notes also included thoughts and interpretations of the research assistants that were shared during both casual conversations and informal post-interview debriefing sessions utilizing principles of reflexivity. [Methods Paragraph 8] |
| 21. | Duration | The interviews lasted between 30-60 minutes each.  [Methods Paragraph 6] |
| 22. | Data saturation | Interviews were conducted until data saturation was achieved. [Methods Paragraph 7] |
| 23. | Transcripts returned | N/A |
| **Domain 3: Analysis and findings** | | |
| Data analysis | | |
| 24. | Number of data coders | Utilizing research observation and notes to contextualize data, one researcher (AL) developed a codebook. [Methods Paragraph 9] |
| 25. | Description of the coding tree | Typed and de-identified interview notes were uploaded to Dedoose a qualitative analysis program, and given codes and sub-codes from the aforementioned codebook. [Methods Paragraph 9] |
| 26. | Derivation of themes | Themes were organized into a cohesive narrative. Key quotes from the translator’s interpretations were identified to provide further evidence for each theme. [Methods Paragraph 9] |
| 27. | Software | Typed and de-identified interview notes were uploaded to Dedoose a qualitative analysis program, and given codes and sub-codes from the aforementioned codebook. [Methods Paragraph 9] |
| 28. | Participant checking | After the initial interviews, twelve of the twenty interviews were identified that required further clarification. These twelve interviews were repeated with the corresponding interviewee to cross-verify that the relevant meaning had been captured and to expand details within the respondents’ interviews. [Methods Paragraph 7] |
| Reporting | | |
| 29. | Quotations presented | Key quotes from the translator’s interpretations were identified to provide further evidence for each theme. Few verbatim quotes were obtained; therefore, the stories told by the interviewees were reported and conveyed through the translator’s paraphrased words. True verbatim translated quotes are indicated by quotation marks. [Methods Paragraph 9] |
| 30. | Data and findings consistent | See quotes in Results section that represent findings. [Results] |
| 31. | Clarity of major themes | See themes presented in Results section. [Results] |
| 32. | Clarity of minor themes | N/A |
