# Supplementary material for: Exploring sources of insecurity for Ethiopian Oromo and Somali women who have given birth in Kakuma Refugee Camp: A Qualitative Study
Source: PLoS Med. 2020 Mar 24;17(3):e1003066. doi: 10.1371/journal.pmed.1003066 (PMC7092956; doi:10.1371/journal.pmed.1003066)
Supplement: S1 Text — (DOCX) [file pmed.1003066.s002.docx]

**Interview Guide Sample Questions**

**Primary themes:**

General worries/feelings of stress

General perceptions of insecurity and safety

Perception of self-health

Pregnancy experience

**Example questions:**

Where are you from? How did you arrive here? How long they have been here?

Ethnic group

Religion

Marital status

Age

Number of children who they live with/ still alive

Location of current home

Have you stayed in the same place in this camp?

How would you describe living here?

What is your family’s main source of income? (Rations, Small business, NGO employment, remittances?)

Are you, yourself, employed/ have a consistent way to earn money?

If not, do you wish to be employed?

If you wish to be employed, what are the barriers to obtaining employment? What would you wish to be doing (and why)?

Who has supported you since you’ve been here / Can you list people you go to when you need **financial/material suppor**t? (some examples may be by providing food, money, ) (Have them free list, and keep probing for more.) (PROBE: family, friends, neighbors?)

Who has supported you since you’ve been here / Can you list people you consider **social support**/ someone you go to talk or when you’re worried/stressed? (Someone to talk to, advice, or childcare) (Have them free list, and keep probing for more.) (PROBE: family, friends, neighbors?)

What forms of assistance was most helpful?  (Probes: some examples may be by providing food, money, someone to talk to, advice, or childcare)

**General worries/feelings of stress**

What are some of your biggest worries on a daily basis? (Free-list as many that come to mind)

(After PROBE: recent examples, stories)

If you could tell me one story of woman on this camp that best describes the experience of a woman on this camp, what would you tell me?

How do you cope with stress? (i.e., praying, sleeping, drinking tea, spending time with family, talking with friends, reading, finding some alone time, seeking medical care or counseling services, etc.) (PROBE: anything you wish you could do?)

If religion is stated: How have your beliefs helped you while living in Kakuma? Have these beliefs changed over time in a way that helps you in your current life situation?

How do you define dignity in each day?

**General perceptions of insecurity (experiences in the house, experiences on the camp)**

Do you feel that Kakuma is a safe camp? Why or why not?

- Day versus Night?

-Safe walking around the camp by yourself?

- Safe in your own home? (From others and even family?)

- Have the conditions of the camp become more or less “safe”?

- Is there a specific part of Kakuma camp that you feel is dangerous?

Where are the main areas that you or other women feel vulnerable or at risk for violence? (Ask to draw map if necessary.)

According to your sense of safety within Kakuma refugee camp, would you feel more or less safe in your home country?

In relation to camp life, how do you feel that women’s lives could be improved and made safer?  What’s one thing you would change?

OR If this refugee camp were perfect, what would it be like?

How do you feel about the authorities when seeking social services?

Have you heard of other women experiencing threats or threatening situations within the camp? Can you describe this situation? (Do they seek aid after?) (PROBE: I have heard sexual assault cases are common, do you think this is true?).

Are there any supports such as clinics or counseling centers that you have utilized? If yes, which services did you utilize?  If yes, how helpful did you find these services to be for you? How did you hear about these services? If not, what services would you wish there were?

**General perception of own health**

How would you describe good health? Do you have this? Do other women you know have this?

Are there things you can do to improve your health? What don’t you have over control to improve health? What are the barriers to obtaining health? Why or why not?

(If not already said: Can you list people you consider social support? How do they help and support you? (Or Who do you feel you can rely on?)

What kinds of problems were there in getting access to proper health care? or traditional medicine?   (PROBE: Are there reasons why you do not seek care at health facilities?)

How do you usually obtain food (i.e. WFP, trading, buying with income?)

What did you eat for breakfast, lunch, and dinner in the past week? Is this a typical meal? If not, can you describe a typical meal in a day? What food is most common in your diet?

**Experience during pregnancies (complications, care taking, access to services).**

How many pregnancies have you had on this camp? __________

How many children have you birthed in this camp? __________

## Have you undergone female genital cutting/circumcision? (Ask only if they feel comfortable?)

Thinking back at how you would describe you early pregnancies, can you tell me what your day-to-day life looked like while pregnant? Did you perform the same daily tasks? Did you seek antenatal care (at least 4 visits)? If so, why and where? If no, why not?

How did you feel while pregnant in Kakuma? Were you healthy?

Did you feel supported by family or the community?

What were your main concerns or worries while pregnant?

Were there any added stressors in your life? Did you feel more protected from violence?

Where did you give birth to your children? Hospital? Home? What were the factors that influenced this decision? (PROBE: birth certificate, C-section)

Did you experience any complications during pregnancy or labor? Would you be willing to share an example experience? Did you seek care? Why or why not?

Can you tell me the general size of your children when they were born. (Ask to see MCH card or show dolls to confirm size and weight).

If you could describe your ideal pregnancy experience, how would you describe it?

If not answered previously, make sure to obtain the following information:

What is your age?

 _____ 18-19 _____20-29 _____50-59 _____60-69

What is your current marital status?

What level of education did you complete?

Primary (Elementary)

Secondary

Post secondary (college or vocational)

Employment Status ______ Employed _____ Self- Employed _____Unemployed

Number of Children (both total and alive): _____

Do you identify with a particular ethnic group, tribe, or clan? _____________ If yes, what is your ethnic/ tribal/ clan identification?___________________

-Religion

-Marital status

-Age

-Number of children who they live with &or number of children still alive

**Debrief:**

Setting of interview:

Body language:

Emerging themes:

Other notes:
